# Microparticles Mediate the Intercellular Regulation of *microRNA-503* and Proline-Rich Tyrosine Kinase 2 to Alter the Migration and Invasion Capacity of Breast Cancer Cells

**DOI:** 10.3389/fonc.2014.00220

**Published:** 2014-08-15

**Authors:** Joyce Gong, Frederick Luk, Ritu Jaiswal, Mary Bebawy

**Affiliations:** ^1^Discipline of Pharmacy, Graduate School of Health, University of Technology Sydney, Sydney, NSW, Australia; ^2^Sydney Medical School and Bosch Institute, The University of Sydney, Sydney, NSW, Australia

**Keywords:** breast cancer, metastasis, microparticles, microarray, microRNA, *microRNA-503*, multidrug resistance, proline-rich tyrosine kinase 2

## Abstract

The successful treatment of cancer is hampered by drug resistance and metastasis. While these two obstacles were once considered separately, recent evidence associates resistance with an enhanced metastatic capacity. However, the underlying mechanisms remain undefined. We previously described the intercellular transfer of drug resistance via submicron vesicles called microparticles (MPs). We now propose that MPs derived from drug-resistant cells are also involved in the intercellular transfer of components to enhance the migration and invasion capacity of cells. Thus, MPs may be a conduit between resistance and metastasis. We used microarray analysis to identify regulatory microRNAs (miRNAs), which contribute to the dissemination of metastatic traits. *miR-503* was downregulated in recipient cells following co-culture with MPs isolated from drug-resistant cells. *miR-503* was inversely associated with metastasis, as demonstrated using wound healing/scratch migration assays and Matrigel^®^-coated transwell invasion assays. Proline-rich tyrosine kinase 2 (PYK2) was upregulated in recipient cells and associated with increased migration and invasion, with these phenotypes being reversed using a pharmacological inhibitor of PYK2 phosphorylation, tyrphostin A9. However, the MP-mediated promotion of metastatic traits was not due to the presence of these effectors in the MP cargo but rather due to down stream effector molecules in these pathways. This is the first demonstration that the role of MPs in trait acquisition extends beyond the direct transfer of vesicle components and also includes transfer of intermediary regulators that induce down stream mediators following transfer to recipient cells. This implicates an expanding role of MPs in cancer pathogenesis.

## Introduction

The development of drug resistance and metastases are significant hindrances to the successful treatment of cancer. Of clinical significance is multidrug resistance (MDR) associated with the overexpression of efflux transporters such as P-glycoprotein (P-gp). P-gp recognizes numerous drug substrates and expels them from cells, maintaining a sublethal intracellular concentration, resulting in treatment failure (1). Recently, P-gp-mediated MDR and the emergence of an enhanced metastatic capacity have been linked in laryngeal carcinoma (2) and breast cancer (3, 4). These studies show that the overexpression of P-gp may be predictive of increased migration and invasive capacity.

We have shown that small plasma membrane-derived vesicles called microparticles (MPs) are involved in the dissemination of MDR (5–8). MPs are vesicles 0.1–1 μm in diameter that are released from cells by membrane budding (9, 10). As such, MPs carry surface antigens, proteins, cytoplasmic and nuclear constituents from the donor cell (5–7, 11, 12). MPs differ from other submicron vesicles such as exosomes in terms of size, shape, content, and origin. Exosomes are smaller (40–80 nm in diameter) and are carried within multivesicular bodies, which fuse with the plasma membrane and release their content (13).

We have shown that MPs can transfer regulatory microRNAs (miRNAs) from donor drug-resistant cancer cells to recipient drug-sensitive cancer cells, effectively re-templating recipient cells to reflect donor cell traits (6–8). miRNAs are small non-coding nucleic acids, 19–25 nucleotides in length, capable of regulating transcriptional and post-transcriptional gene expression by pairing with the 3′-untranslated region (UTR) of target mRNAs (14). Herein, we examined the involvement of MPs derived from drug-resistant breast cancer cells in conferring an enhanced migration and invasion capacity to recipient cells. In particular, we found a MP-mediated involvement of *miR-503* and proline-rich tyrosine kinase 2 (PYK2).

The regulation of *miR-503* has been previously shown to be involved in the development of drug resistance and metastatic traits, with reduced levels of *miR-503* being identified in drug-resistant cells (15) and highly metastatic cells (16). One pathway by which *miR-503* acts as a tumor suppressor is via downregulation of phosphatidylinositol 3-kinase (PI3K)/AKT signaling (17). The PI3K/AKT signaling pathway plays an important role in the progression of breast cancer (18, 19). As a major pathway, PI3K/AKT signaling is not only regulated by *miR-503* but also by other factors including the focal adhesion kinase PYK2. PYK2, also known as related adhesion focal tyrosine kinase (RAFTK) or protein tyrosine kinase 2 beta (PTK2B), is a member of the focal adhesion kinase subfamily of cytoplasmic tyrosine kinases (20). Elevated levels of PYK2 have been associated with enhanced migration and invasion via activation of the PI3K/AKT signaling pathway (21). Here, we have investigated the role of MPs derived from drug-resistant cells in not only disseminating the drug resistance trait but also in the regulation of both *miR-503* and PYK2 in recipient cells to promote migration and invasion. Therefore, MPs may provide a conduit between drug resistance and an enhanced migration and invasion capacity in cancer via activation of PI3K/AKT signaling.

## Materials and Methods

### Cell culture

The drug-sensitive human breast adenocarcinoma cell line MCF-7 and its drug-resistant subline MCF-7/Dx were cultured as previously described (6, 7). The drug-sensitive human acute lymphoblastic leukemia cell line CCRF-CEM (designated CEM for simplicity) and its drug-resistant variant VLB_100_ were also used as previously described (6, 7). All cells were cultured with RPMI 1640 culture medium supplemented with 10% fetal bovine serum (FBS) at 37°C with 5% CO_2_.

### Microparticle isolation

Microparticles were isolated as previously described (22). Briefly, cell culture supernatants were centrifuged at 500 × *g* for 5 min to pellet cells and large debris. The supernatant was centrifuged again at 15,000 × *g* for 1 h at 17°C to pellet MPs. The MP pellet was resuspended in serum-free media and centrifuged at 2,000 × *g* for 1 min to remove small debris and the remaining supernatant centrifuged again at 18,000 × *g* for 30 min at 17°C to pellet MPs. The isolated MP fraction was validated by flow cytometric analysis (LSRII flow cytometer, BD Biosciences, Sydney, NSW, Australia) following V450 Annexin V labeling (BD Biosciences) and by size as previously described (5). The total protein content of the MP fraction was determined using the Qubit^®^ protein assay (Life Technologies, Melbourne, VIC, Australia) following the manufacturer’s recommendation. MPs isolated from MCF-7 and MCF-7/Dx cells were designated MCFMPs and DxMPs, respectively. MPs isolated from CEM and VLB_100_ cells were designated CEMMPs and VLBMPs, respectively.

### Co-culture conditions

1 × 10^5^ drug-sensitive MCF-7 cells were co-cultured with 100 μg MCFMPs or DxMPs in culture medium for 4 h. Cells were centrifuged at 500 × *g* for 5 min and washed twice with phosphate-buffered saline (PBS) to remove MPs. Cells were then analyzed as outlined below.

### Migration and invasion assays

In assessing cell migration, a wound healing/scratch migration assay was used. Confluent cells grown in six-well culture plates (Corning, Sydney, NSW, Australia) were scratched using a sterile 10 μL pipette tip and washed twice to remove detached cells and debris. Cell migration was monitored at 0 and 48 h post-scratch. The closure of the wound after 48 h was measured using ImageJ software and the percentage wound closure relative to that measured at 0 h was calculated. As both MCF-7 and MCF-7/Dx cell lines have a doubling time of 24 h (data not shown), the extent of wound closure is reflective of the migration capacity of cells rather than the result of difference in doubling time.

The invasive capacity of cells was determined using transwell inserts (24-well 6.5 mm insert, pore size 8 μm, Corning). Inserts were coated with Matrigel^®^ basement membrane (BD Biosciences) before cells were seeded to the upper chamber in serum-free RPMI 1640. RPMI 1640 with 10% FBS was added to the lower chamber as a chemoattractant. After incubation for 48 h at 37°C and 5% CO_2_, the cells that had emerged from the underside of the Matrigel^®^-coated inserts were fixed and stained with 0.5% crystal violet in 20% methanol for 10 min.

### Identification of microRNAs associated with the acquisition of drug resistance

Total RNA was pooled from duplicate experiments and extracted using the Tri Reagent method (Molecular Research Center, Cincinnati, OH, USA). RNA integrity was determined using the Agilent RNA 6000 Nano kit and Agilent 2100 Bioanalyzer (Agilent Technologies, Melbourne, VIC, Australia). Total RNA was quantitated by Nanodrop 1000 spectrophotometer (Thermo Fisher Scientific, Melbourne, VIC, Australia). miRNA microarray analysis was performed with internal replicates for each probe on the Affymetrix GeneChip^®^ miRNA Array (Affymetrix, Santa Clara, CA, USA) by the Australian Genome Research Facility Ltd. (Melbourne, VIC, Australia) as previously described by us (7). Microarray data can be accessed in NCBI’s Gene Expression Omnibus through GEO Series accession number GSE34560 (http://www.ncbi.nlm.nih.gov/geo/query/acc.cgi?acc=GSE34560). Data were processed to include only human miRNAs with *p*-values < 0.06. We compared the miRNA expression profiles from samples derived from: (A) drug-resistant cells (MCF-7/Dx or VLB_100_), and (B) drug-sensitive cells co-cultured with MPs derived from drug-resistant cells (MCF-7 + DxMPs or CEM + VLBMPs) with (C) drug-sensitive cells (MCF-7 or CEM). miRNAs with a fold change greater than 1.5 were identified and used for subsequent analysis. The profile of differentially expressed miRNAs as determined by comparison analysis was represented as a Venn diagram with the final list of miRNAs representative of miRNAs that were differentially expressed in drug-resistant cells and co-cultured cells relative to drug-sensitive cells.

### Transfection of *miR-503* antagomiR and pre-miR

We sought to determine the functional consequences of MP-transferred *miR-503* on recipient cells. mirVana^®^ *miR-503* antagomiR, mirVana^®^ *miR-503* pre-miR, and its scrambled control (Life Technologies) were used to study the effect of *miR-503* on the migration and invasion capacity of cells. MCF-7 and MCF-7/Dx cells were transfected for 24 h with 40 nM *miR-503* antagomiR, pre-miR or the scrambled control using Lipofectamine 2000 (Life Technologies) according to the manufacturer’s instructions. MCF-7 and MCF-7/Dx cells were assessed for their migration and invasion capacity using migration and invasion assays as described above.

### Proline-rich tyrosine kinase 2 expression

Proline-rich tyrosine kinase 2 is also associated with the promotion of migration and invasion. To determine the expression levels of PYK2 in MCF-7 cells, MCF-7 cells co-cultured with DxMPs, MCF-7/Dx cells, and DxMPs, samples were lysed using CelLytic™ M Cell Lysis reagent (Sigma-Aldrich, Sydney, NSW, Australia) with 1% (v/v) protease inhibitor cocktail (Sigma-Aldrich) according to the manufacturer’s recommendation. The lysates were centrifuged at 10,000 × *g* for 10 min at 4°C and protein content quantified by Qubit^®^ protein assay (Life Technologies). Total protein was separated by electrophoresis using a 4–12% NuPAGE™ Bis-Tris gel (Life Technologies) and PYK2 was detected by Western blot using anti-PYK2 (clone 5E2) mouse monoclonal antibody. Anti-β-actin (clone AC-74) antibody was used as an internal loading control.

The presence of the *Pyk2* gene transcript was assessed by quantitative real time PCR (qRT-PCR). Briefly, total RNA was extracted from samples by the Tri Reagent method (Molecular Research Center) according to the manufacturer’s instructions. cDNA was synthesized using the Advantage RT-for-PCR Kit (Clontech Laboratories, Mountain View, CA, USA) on the Eppendorf MasterCycler Gradient (Eppendorf, Hauppauge, NY, USA). The following primers (Sigma-Aldrich) were used: *Pyk2* forward: 5′-CCCAGCCGACCTAAGTACAG-3′; reverse: 5′-CACACAGACCCTCAGGAACC-3′. The following housekeeping primers (Sigma-Aldrich) were used: *GAPDH* forward: 5′-TGCCAAATATGATGACATCAAGAA-3′; reverse: 5′-GGAGTGGGTGTCGCTGTTG-3′. SYBR Green (Takara Bio Inc., Mountain View, CA, USA) qRT-PCR amplifications were performed on the Eppendorf Realplex 2 MasterCycler ep gradient S (Eppendorf). The Ct data of each sample were collected automatically. The ΔCt of each group was calculated using the following formula: ΔCt = *Pyk2* Ct - GAPDH Ct. The relative expression levels were calculated using ΔΔCt = 2^−ΔCt^ and expressed as fold differences from drug-sensitive MCF-7 cells: ([ΔΔCt of sample ÷ ΔΔCt of MCF-7 cells] × 1).

### Statistical analysis

Data were analyzed using one-way analysis of variance (ANOVA) with a *post hoc* Tukey’s multiple comparison test using the Graph Pad Prism software. *p*-values < 0.05 were accepted as statistically significant.

## Results

### Microparticles isolated from MDR cells enhance the migration and invasion capacity of recipient cells

**Figure 1:**
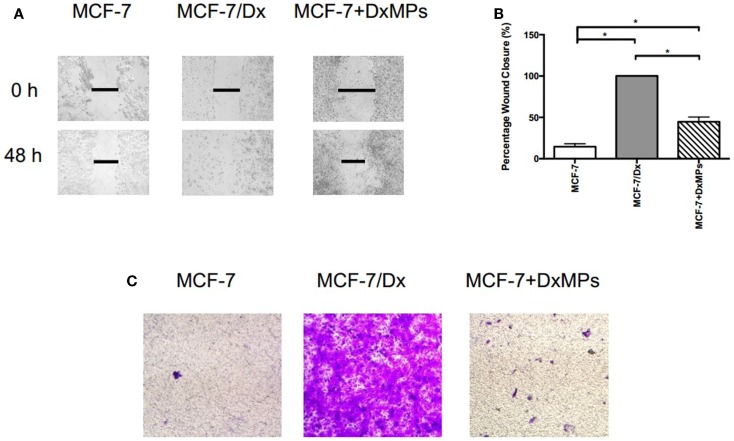
**Microparticles mediate the promotion of migration and invasion in breast cancer**. **(A)** Migratory capacity of breast cancer cells. Wound healing/scratch migration assays of MCF-7 cells, MCF-7/Dx cells, and MCF-7 cells co-cultured with DxMPs demonstrate the migratory capacity of each cell type. Closure of the wound was assessed after 48 h. Data was representative of at least three independent experiments. **(B)** Histograms showing the percentage wound closure in breast cancer cells. Percentage wound closure observed in (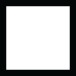): MCF-7 cells, (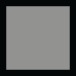): MCF-7/Dx cells, and (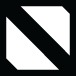): MCF-7 cells co-cultured with DxMPs. Data represent the mean ± SEM of at least three independent experiments. *p < 0.05. **(C)** Invasive capacity of breast cancer cells. Transwell insert invasion by MCF-7 cells, MCF-7/Dx cells, and MCF-7 cells co-cultured with DxMPs. Invasion of cells through a Matrigel^®^ layer to the underside of transwell membranes was assessed after 48 h. Data was representative of at least three independent experiments.

To determine the role of MPs in the regulation of migration and invasion, we co-cultured drug-sensitive MCF-7 breast cancer cells with MPs isolated from drug-resistant MCF-7/Dx cells (DxMPs) and assessed their effects on cell migration and invasive capacity. A wound was created in confluent layers of MCF-7, MCF-7/Dx, and co-cultured cells (MCF-7 + DxMPs). Closure of the wound was imaged at the time the wound was created (0 h) and after 48 h (Figure 1A). ImageJ analysis of the size of the wound allowed for calculation of the percentage of wound closure after 48 h (Figure 1B). On average, the size of the wound in MCF-7 cells closed by 14%, indicating a low migratory capacity for this cell line. In MCF-7/Dx cells, the wound closed completely after 48 h. When co-cultured with DxMPs, the wound in the resultant population closed by 45% during the same timeframe, indicating that DxMPs increased the migratory capacity of MCF-7 cells, which was reflective of that observed with the donor MCF-7/Dx cells.

In addition, MPs enhanced the invasive capacity of recipient cells. The invasion of cells through a Matrigel^®^ layer to the underside of a transwell membrane insert were observed (Figure 1C). MCF-7 cells had a low invasive capacity, as evidenced by the low number of cells that invaded the Matrigel^®^ layer. In contrast, MCF-7/Dx cells readily invaded to almost completely cover the underside of the transwell. Consistent with that observed in the migration assay, following MP co-culture of MCF-7 cells, an intermediate number of co-cultured cells invaded the Matrigel^®^ layer. Therefore, DxMPs increased both the migratory and invasive capacity of MCF-7 cells to reflect that observed for donor MCF-7/Dx cells.

### Identification of microRNAs associated with the acquisition of drug resistance

**Figure 2:**
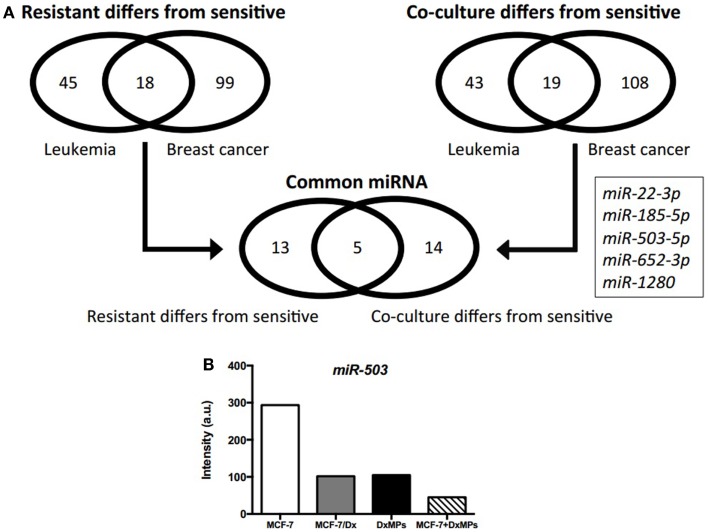
**Microarray analysis of expressed miRNAs**. **(A)** Profile of differentially expressed miRNAs as determined by comparison analysis. Venn diagrams illustrate miRNAs that were differentially expressed in drug-resistant cells compared to drug-sensitive cells and miRNAs that were differentially expressed in co-cultured cells compared to drug-sensitive cells. Five miRNAs were common across both cancers that were differentially expressed in drug-resistant and co-cultured cells compared to drug-sensitive cells. **(B)** Expression of *miR-503* as determined by microarray analysis. Microarray expression levels of *miR-503* in (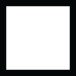): MCF-7 cells, (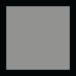): MCF-7/Dx cells, (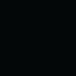): DxMPs, and (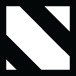): MCF-7 cells co-cultured with DxMPs.

After identifying miRNAs that were packaged in MPs and delivered to recipient cells (7), we employed different selection criteria to the microarray data to identify miRNAs associated with the acquisition of drug resistance in recipient cells. Specifically, we compared the differentially expressed miRNAs in co-cultured cells to those in drug-resistant donor cells. Venn diagrams show that 63 (leukemia) and 117 (breast cancer) miRNAs were differentially expressed in drug-resistant cells compared to drug-sensitive cells, with 18 of these miRNAs being common to both cancer cell types (Figure 2A). Similarly, 62 (leukemia) and 127 (breast cancer) miRNAs were differentially expressed in co-cultured cells compared to drug-sensitive cells, with 19 of these miRNAs being common to both cancer cell types. As a result, five miRNAs were identified as being potentially associated with the MP-mediated acquisition of MDR: *miR-22-3p*, *miR-185-5p*, *miR-503-5p*, *miR-652-3p*, and *miR-1280*. Of these, *miR-503-5p* (also known as *miR-503*) was selected by us for further investigation due to its previous association in suppressing the metastatic function of cells (16). Microarray analysis showed that *miR-503* expression was highest in MCF-7 cells, with low levels in MCF-7/Dx cells, DxMPs, and MCF-7 cells co-cultured with DxMPs (Figure 2B). Therefore, we sought to transfect cells with a *miR-503* antagomiR or *miR-503* pre-miR to validate the effects of *miR-503* on the migration and invasion capacity of recipient cells.

### Perturbation of *miR-503* altered the migration and invasive capacity of breast cancer cells

**Figure 3:**
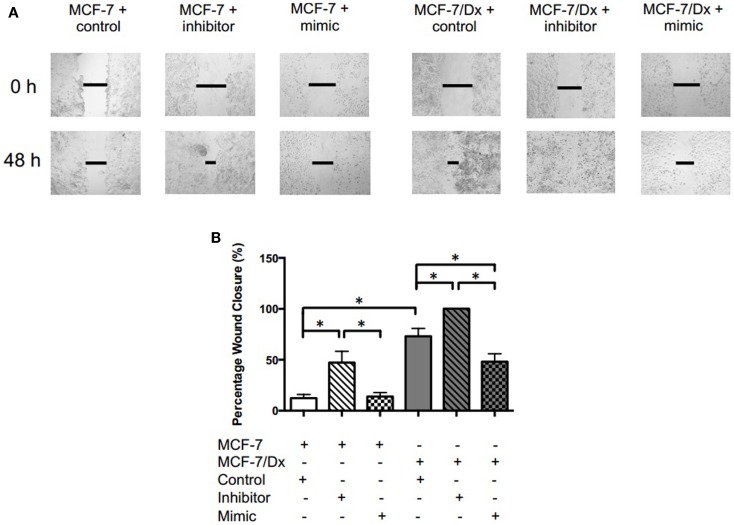
***miR-503* is involved in regulating the migratory capacity of breast cancer cells**. **(A)** Migratory capacity of transfected breast cancer cells. Wound healing/scratch migration assays of MCF-7 cells and MCF-7/Dx cells demonstrate the migratory capacity of each cell type and the effect of the inhibition or mimicry of *miR-503* on this capacity. Closure of the wound was assessed after 48 h. Data was representative of at least three independent experiments. **(B)** Histograms showing percentage wound closure in transfected breast cancer cells. Percentage wound closure observed in (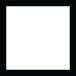): MCF-7 cells transfected with the scrambled control, (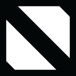): MCF-7 cells transfected with the *miR-503* antagomiR, (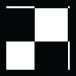): MCF-7 cells transfected with the *miR-503* pre-miR, (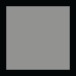): MCF-7/Dx cells transfected with the scrambled control, (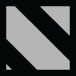): MCF-7/Dx cells transfected with the *miR-503* antagomiR, and (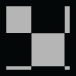): MCF-7/Dx cells transfected with the *miR-503* pre-miR. Data represent the mean ± SEM of at least three independent experiments. *p < 0.05.

Closure of the wound created in confluent layers of MCF-7 and MCF-7/Dx cells previously transfected with a *miR-503* antagomiR, pre-miR, or the scrambled control was assessed (Figure 3A). The percentage wound closure after 48 h was then calculated (Figure 3B). On average, the size of the wound in MCF-7 cells transfected with the scrambled control closed by 12%. In contrast, MCF-7 cells transfected with the *miR-503* antagomiR showed an increased percentage wound closure at 47%. When transfected with the miR-503 pre-miR, the percentage wound closure remained at similar levels to that observed following transfection with the scrambled control at 14%. Drug-resistant MCF-7/Dx cells transfected with the scrambled control had wounds that closed by 73%. Their transfection with the *miR-503* antagomiR resulted in complete closure of the wound while transfection with the *miR-503* pre-miR reduced the wound closure to 48%. The inhibition of *miR-503* thus resulted in increased migration of MCF-7 and MCF-7/Dx cells, while mimicry of *miR-503* reduced migration.

**Figure 4:**
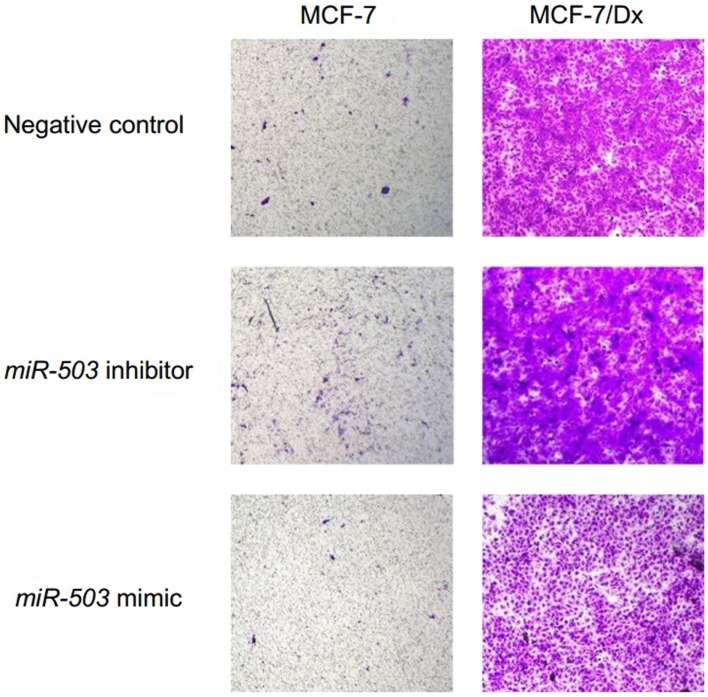
***miR-503* is involved in regulating breast cancer invasive capacity**. Transwell insert invasion by MCF-7 cells and MCF-7/Dx cells demonstrate the invasive capacity of each cell type and the effect of the inhibition or mimicry of *miR-503* on this capacity. Invasion of cells through a Matrigel^®^ layer to the underside of transwell membranes was assessed after 48 h. Data was representative of at least three independent experiments.

Moreover, *miR-503* was involved in the invasive capacity of MCF-7 and MCF-7/Dx cells. When MCF-7 cells were transfected with the scrambled control, 12 cells per field of vision were present on the underside of the transwell membrane (Figure 4). This is consistent with MCF-7 cells having a low invasive capacity. However, once MCF-7 cells were transfected with the *miR-503* antagomiR, 107 cells per field of vision invaded the Matrigel^®^ layer, emerging on the underside of the transwell membrane. The number of invasive cells decreased to that observed with the scrambled control when MCF-7 cells were transfected with the *miR-503* pre-miR. As observed in the migration assay, MCF-7/Dx cells transfected with the scrambled control invaded through to the underside of the membrane to completely cover it (Figure 4). Again, transfection with the *miR-503* antagomiR resulted in increased invasion of MCF-7/Dx cells, while transfection with the *miR-503* pre-miR reduced invasion. Overall, the inhibition of *miR-503* resulted in increased invasion of MCF-7 and MCF-7/Dx cells, while the mimicry of *miR-503* reduced their invasion.

### Microparticles isolated from MDR cells mediate PYK2 expression in recipient cells

**Figure 5:**
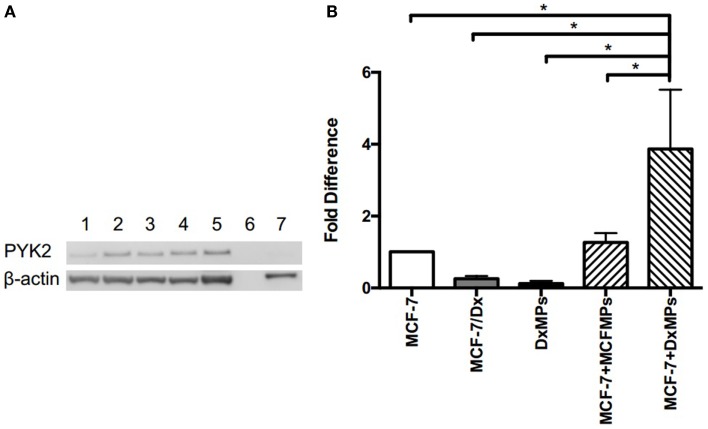
**Upregulation of PYK2 protein and transcript following co-culture with DxMPs**. **(A)** PYK2 protein expression as determined by Western blotting. Total lysates of MCF-7 cells (lane 1), MCF-7 cells co-cultured with DxMPs (lanes 2, 3, and 4), MCF-7/Dx cells (lane 5), and DxMPs (lane 7) were examined for PYK2 expression by Western blotting using the PYK2 (clone 5E2) mAb. β-actin was used as an internal control. Data was representative of at least three independent experiments. **(B)** *Pyk2* gene transcript expression as determined by qRT-PCR. *Pyk2* gene transcript in (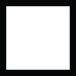): MCF-7 cells, (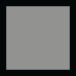): MCF-7/Dx cells, (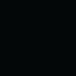): DxMPs, (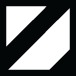): MCF-7 cells co-cultured with MCFMPs, and (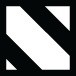): MCF-7 cells co-cultured with DxMPs. Values are expressed as the fold difference relative to MCF-7 cells using *GAPDH* as the endogenous control. Data represent the mean ± SEM of at least three independent experiments. *p < 0.05.

In addition to *miR-503* expression, the tyrosine kinase PYK2 is also implicated in promoting cancer metastasis. Western blot analysis was used to detect the presence of PYK2 in MCF-7 cells, co-cultured cells, MCF-7/Dx cells, and DxMPs. PYK2 was identified at 116 kDa (Figure 5A). MCF-7 cells expressed low levels of PYK2 (lane 1). However, once they were co-cultured with DxMPs, there was an increase in PYK2 expression (lanes 2, 3, and 4). Donor MCF-7/Dx cells expressed the most PYK2 (lane 5). However, their MPs expressed negligible levels of PYK2 (lane 7).

We next sought to determine the expression profile of the *Pyk2* gene transcript using qRT-PCR. The level of *Pyk2* in each sample was expressed as a fold difference relative to that present in MCF-7 cells (Figure 5B). MCF-7/Dx cells expressed 3.8-fold less *Pyk2* than MCF-7 cells, with their MPs expressing 8.2-fold less. Although DxMPs expressed very little of the transcript, once they were co-cultured with MCF-7 cells, the resultant recipient cell population showed a dramatic 3.9-fold increase in *Pyk2* expression. A co-culture with MCFMPs (MPs derived from MCF-7 cells) was used as a control, and resulted in an insignificant 1.27-fold increase in *Pyk2* expression. Therefore, the increased *Pyk2* expression in co-cultured cells was not due to a membrane-induced artifact following exposure to MPs, but rather to an interaction specific to DxMPs, independent of their own *Pyk2* expression levels.

### Increased PYK2 expression promotes migration and invasion

**Figure 6:**
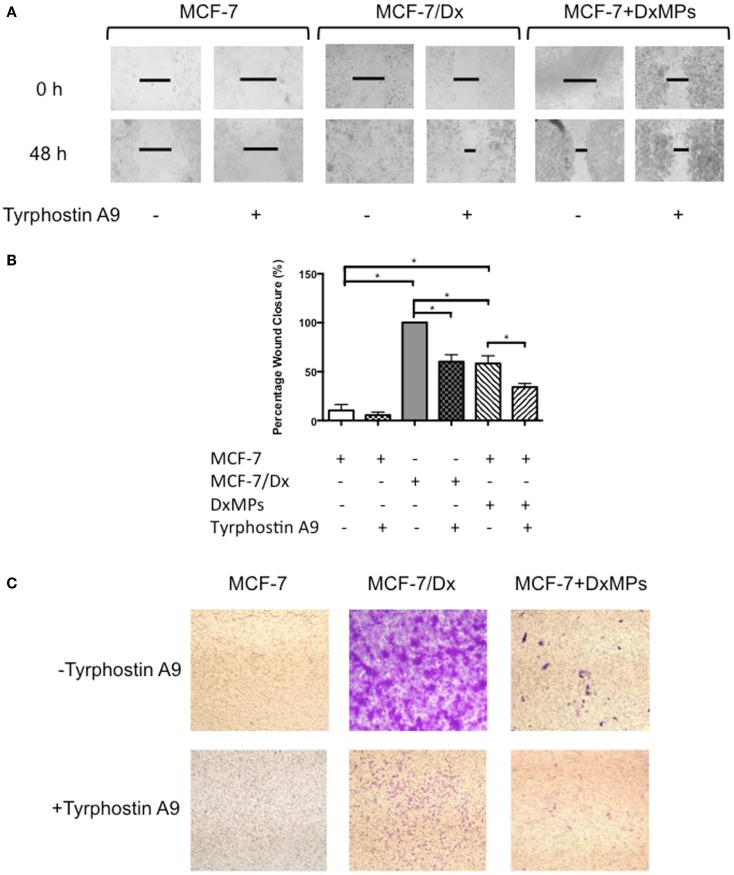
**Inhibition of PYK2 phosphorylation is associated with decreased migratory capacity**. **(A)** Migratory capacity of breast cancer cells. Wound healing/scratch migration assays of MCF-7 cells, MCF-7/Dx cells, and co-cultured cells demonstrate the migration capacity of each cell type and the effect of the PYK2 phosphorylation inhibitor tyrphostin A9 on this capacity. Cells were incubated without or with tyrphostin A9 and closure of the wound was assessed after 48 h. Data was representative of at least three independent experiments. **(B)** Histograms showing the percentage wound closure in breast cancer cells. The percentage wound closure observed in (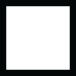): MCF-7 cells without tyrphostin A9, (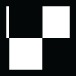): MCF-7 cells with tyrphostin A9, (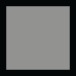): MCF-7/Dx cells without tyrphostin A9, (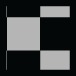): MCF-7/Dx cells with tyrphostin A9, (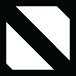): MCF-7 cells co-cultured with DxMPs without tyrphostin A9, and (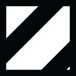): MCF-7 cells co-cultured with DxMPs with tyrphostin A9. Data represent mean ± SEM of at least three independent experiments. *p < 0.05. **(C)** Inhibition of PYK2 phosphorylation is associated with decreased invasive capacity. Transwell insert invasion by MCF-7 cells, MCF-7/Dx cells, and co-cultured cells demonstrate the invasive capacity of each cell type and the effect of tyrphostin A9 on this capacity. Cells were incubated without or with tyrphostin A9 and the invasion of cells through a Matrigel^®^ layer to the underside of transwell membranes was assessed after 48 h. Data was representative of at least three independent experiments.

In order to determine the functional effect of PYK2 on migration and invasion, wound healing/scratch migration and transwell invasion assays were again conducted. Cells were treated with 10 μM of the PYK2 phosphorylation inhibitor tyrphostin A9 to determine whether functional PYK2 had effects on migration and invasion. The closure of the wound created in confluent layers of MCF-7 cells, MCF-7/Dx cells, and MCF-7 cells co-cultured with DxMPs was assessed following treatment with tyrphostin A9 or with the solvent control (Figure 6A). Closure of the wound was imaged after 48 h. ImageJ analysis of the size of the wound allowed for calculation of the percentage of wound closure after 48 h (Figure 6B). On average, the wound in MCF-7 cells without tyrphostin A9 treatment closed by 10%. This decreased to 5% when MCF-7 cells were treated with tyrphostin A9. As expected, MCF-7/Dx cells without tyrphostin A9 showed complete closure of the wound. However, once they were treated with tyrphostin A9, this decreased to 60%. When MCF-7 cells were co-cultured with DxMPs, the migratory capacity increased to reflect that of the donor cell, with the wound in co-cultured cells without tyrphostin A9 closing by 58%. Again, treatment with tyrphostin A9 reduced the percentage wound closure to 34%. Therefore, inhibition of the phosphorylation of PYK2 resulted in decreased migration.

Consistent with the results in the migration assay, the transwell invasion assay demonstrated that treatment with tyrphostin A9 reduced the invasion of cells through a Matrigel^®^ layer. With and without tyrphostin A9 treatment, MCF-7 cells were trapped within the Matrigel^®^ layer and did not invade on the underside of the transwells (Figure 6C). In contrast, while MCF-7/Dx cells strongly invaded the Matrigel^®^ layer, when treated with tyrphostin A9, fewer cells penetrated to the underside of the transwell membrane. Similar to the migration assay, MCF-7 cells co-cultured with DxMPs displayed an intermediate invasive capacity relative to recipient MCF-7 cells and donor MCF-7/Dx cells, with several cells invading the Matrigel^®^ layer. As with MCF-7/Dx cells, treatment with tyrphostin A9 reduced the ability of co-cultured cells to invade. Therefore, inhibition of PYK2 phosphorylation using tyrphostin A9 reduced the invasive capacity of cells.

### *miR-494* and *miR-330-3p* regulate *Pyk2* expression

**Figure 7:**
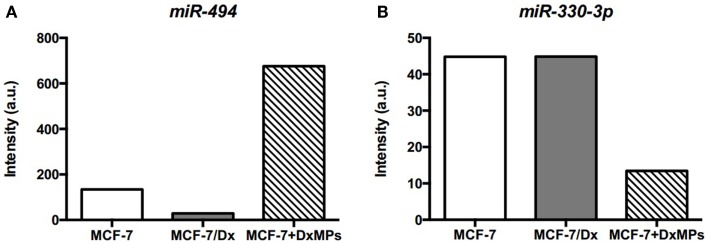
**Expression of miRNAs as determined by microarray analysis**. Expression levels of **(A)** *miR-494* and **(B)** *miR-330-3p* in cells. Microarray expression levels of miRNAs in (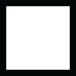): MCF-7 cells, (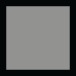): MCF-7/Dx cells, and (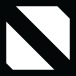): MCF-7 cells co-cultured with DxMPs.

Microarray analysis of MCF-7 cells, MCF-7/Dx cells, and MCF-7 cells co-cultured with DxMPs showed that *miR-494* was highly expressed in co-cultured cells compared to the parental cells (Figure 7A). Conversely, *miR-330-3p* was expressed in low levels in co-cultured cells (Figure 7B). Using the online database miRDB for miRNA target prediction and functional annotations (http://mirdb.org/miRDB), we identified *Pyk2* as a target of these miRNAs. Therefore, they may be a means by which MPs promote the expression of *Pyk2*.

## Discussion

Although the development of drug resistance and metastases are both major considerations in the clinical treatment of cancer, they have only recently been studied together. The drug-resistant MCF-7/Dx cells used in this study are cells that have been derived from drug-sensitive MCF-7 cells by incremental exposure to doxorubicin (23). In transforming these cells to a drug-resistant phenotype, MCF-7/Dx cells have also gained characteristics that are reflective of a mesenchymal phenotype (24, 25). Compared to MCF-7 cells, MCF-7/Dx cells grow in a disperse manner rather than in clusters, have lost the expression of E-cadherin while gaining expression of N-cadherin, which is a hallmark of epithelial to mesenchymal transition (EMT) (24), are vimentin-positive, and are highly invasive (25).

We have shown that MPs disseminate drug resistance (5–7, 12). Here, we have elucidated the larger role of MPs derived from drug-resistant breast cancer cells in disseminating components to promote the migration and invasion capacity of recipient cells. This is the first demonstration that MPs derived from drug-resistant cells also confer an enhanced migration and invasion capacity. Therefore, MPs may serve as a conduit between drug resistance and migration and invasion. To identify the possible mechanisms behind this relationship, we studied the differential expression of miRNAs in our breast cancer model and the role of MPs in their distribution between cells.

Selection criteria identified miRNAs that were acquired by co-cultured recipient cells identical to those present in donor drug-resistant cells. This approach aided in identifying five miRNAs that were associated with the dissemination of MDR by MPs. Of the five miRNAs, *miR-503* was further explored as it has been previously implicated in drug resistance and migration and invasion capacity in cancer (15, 16).

In this study, we describe a role for MPs in the regulation of *miR-503* and transfer of an enhanced migration and invasion capacity from drug-resistant to drug-sensitive cells. We observed an inverse relationship between miR-503 and migration and invasion capacity. Specifically, we observed high *miR-503* levels in drug-sensitive MCF-7 cells, which have a low migration and invasion capacity. In contrast, lower levels of *miR-503* were observed in drug-resistant MCF-7/Dx cells, which have a higher migration and invasion capacity. Transfection with a *miR-503* antagomiR increased the migration and invasion of both cells (Figures 3 and 4). In contrast, transfection with a *miR-503* pre-miR reduced migration and invasion (Figures 3 and 4). Moreover, upon co-culture with DxMPs, recipient MCF-7 cells expressed low levels of *miR-503* and had a greater migration and invasion capacity, similar to donor cells. Therefore, both the inhibition of *miR-503* by transfection and following co-culture with DxMPs, reduced the levels of *miR-503* in recipient cells and increased their migration and invasion capacity. Therefore, it is highly likely that DxMPs carry and transfer intermediates that result in the inhibition of *miR-503* in recipient cells, promoting migration and invasion.

Activation of the NF-κB pathway has been shown to suppress the expression of *miR-503* in epithelial cells (26). Furthermore, NF-κB signaling is a significant aspect of the development of drug resistance in cancer, such that drugs that inhibit the NF-κB pathway result in reversal of the MDR phenotype (27–29). Moreover, NF-κB has been associated with the promotion of migration and invasion in breast cancer (30). Therefore, it is feasible that MPs enable the activation of NF-κB signaling and its subsequent suppression of *miR-503* to facilitate both the development of drug resistance and the enhancement of metastatic traits.

Reduced levels of *miR-503* were observed in cisplatin-resistant non-small cell lung cancer (NSCLC) cells, while its overexpression re-sensitized these cells to cisplatin via modulation of the apoptosis regulator Bcl-2 (15). Furthermore, *miR-503* directly targeted and repressed the Fanconi anemia complementation group A protein (FANCA) gene to sensitize NSCLC cells to cisplatin treatment (31). In addition to its role in drug resistance, *miR-503* also serves as a tumor suppressor. Overexpression of *miR-503* inhibited the migration and invasion of the highly invasive hepatocellular carcinoma (HCC) cell line, HCCLM3 (16), induced G1 cell cycle arrest and reduced the proliferation of HCCLM3 cells (16, 32), an acute myeloid leukemia cell line (33), human glioblastoma multiforme (GBM) cell lines (17), osteosarcoma cells and colon cancer cells (34), and suppressed the proto-oncogene cyclin D1 (CCND1) in a human head and neck carcinoma cell line (35). Furthermore, *miR-503* was shown to initiate its tumor suppressive activity via regulation of PI3K/AKT signaling by inhibiting AKT activation (17) and suppressing the PI3K p85 subunit (36).

PI3K/AKT signaling is a major contributor to the aberrant growth and rapid spread of cancer and therefore holds significant interest in the field of cancer therapy (37, 38). Similar to its role in suppressing *miR-503*, and thereby upregulating PI3K/AKT signaling, MPs may further perpetuate this pathway by mediating the expression of PYK2. High levels of PYK2 were associated with poor survival and metastasis in HCC via activation of the PI3K/AKT pathway by PYK2-dependent phosphorylation of AKT, which was reversed by the PI3K/AKT inhibitor LY294002 (21). PYK2 has been further implicated in cancer cell migration and invasion (39), with elevated levels of PYK2 correlating with the progression of HCC (40, 41) and astrocytomas (42). Increased PYK2 expression was observed in early and advanced breast cancer compared to benign and normal breast tissue (39), and in pulmonary metastases, with the inhibition of PYK2 resulting in reduced tumor development and metastasis (20). Furthermore, the expression of PYK2 was found to increase the invasive potential of MDA-MB-435 and MCF-7 breast cancer cells via activation of Src and the MAP kinase pathway (43). The overexpression of PYK2 also induced EMT in Hep3B HCC cells, promoting cell motility and invasiveness for enhanced metastasis (44). This may be due to the involvement of PYK2 in upregulating the phosphorylation and localization of the transcription factor Hic-5, which regulates EMT (44). Moreover, these effects were downregulated upon suppression of PYK2 in metastatic MHCC97L HCC cells (44).

In agreement with the above studies, we observed low PYK2 in lowly metastatic MCF-7 cells and high expression in highly metastatic MCF-7/Dx cells. Once MCF-7 cells were co-cultured with DxMPs, there was an increase in PYK2. However, there were no detectable amounts of PYK2 in the DxMPs themselves. Similarly, when we investigated expression of the *Pyk2* gene transcript, there were very low levels in both DxMPs and the donor MCF-7/Dx cells. However, there was a 3.9-fold increase of *Pyk2* in co-cultured cells compared to MCF-7 cells (Figure 5). Given that DxMPs did not carry either the protein or the gene transcript, there must be other intermediates contained within the vesicle cargo by which DxMPs upregulate PYK2 and *Pyk2* expression independent of the PYK2 load itself. Therefore, the acquisition of protein and transcript in recipient cells was independent of their presence in the MP cargo. This expands our understanding of the intercellular transfer of cellular constituents by microvesicles until now, the cellular constituents acquired were present in the donor cells and/or their MPs. This introduces for the first time a capacity to readily establish proteomic and transcriptional variants independent of that which is directly contained within the MP cargo, possibly via the transfer of intermediate regulators.

For instance, microarray analysis showed that *miR-494* was highly expressed in co-cultured cells compared to MCF-7 and MCF-7/Dx cells (Figure 7A). *miR-494* is predicted to target the focal adhesion kinase family interacting protein of 200 kDa (FIP200) gene. FIP200, also known as retinoblastoma coiled coil protein 1 (RB1CC1), has been shown via co-transfection, binding, and co-immunoprecipitation assays to bind directly to the kinase domain of PYK2 and inhibit its activity (45). Naturally, the downregulation of FIP200 increased the activity of PYK2 (46). Therefore, the high level of *miR-494* may be involved in downregulating the FIP200 gene, enabling the overexpression of *Pyk2*, and subsequent enhancement of metastatic traits in co-cultured cells.

Concurrently, *miR-330-3p* was detected in low levels in co-cultured cells compared to MCF-7 and MCF-7/Dx cells (Figure 7B). Being a regulator of *Pyk2*, it correlated with the overexpression of *Pyk2* in co-cultured cells. Therefore, the observed downregulation of *miR-330-3p* in co-cultured cells may also contribute to the PYK2-mediated promotion of migration and invasion. In this way, regulatory miRNAs may be a means by which DxMPs alter PYK2 protein and gene transcript expression in recipient cells independent of the protein or gene transcript ultimately implicated downstream in the recipient cells.

Another pathway by which DxMPs may upregulate PYK2 in co-cultured cells is via expression of CD44. CD44 is a cell adhesion molecule that has been shown to induce PYK2 phosphorylation via the activation of Src family kinases that phosphorylate PYK2, leading to its increased activity (47, 48). We have recently shown that while CD44 is not expressed in parental MCF-7 cells, it is present in donor MCF-7/Dx cells and their MPs (12). However, upon co-culture with DxMPs, MCF-7 cells did not acquire CD44 (12). Therefore, it is likely that DxMPs do not directly transfer CD44 or PYK2, but rather that the interaction of CD44 found on DxMPs stimulates the activation of PYK2 on recipient cells. Moreover, the upregulation of PYK2 only occurred upon co-culture with DxMPs and not MCFMPs. This lends further support to the link between MDR and the metastatic trait, that only MPs derived from drug-resistant cells and not MPs from drug-sensitive cells could increase the expression of PYK2, resulting in the promotion of migration and invasion in recipient cells.

## Conclusion

MPs not only mediate the intercellular transfer of P-gp-mediated MDR but are now also implicated in promoting migration and invasion in recipient cells, potentially providing a link between these two deleterious traits. Such mechanisms include the regulation of *miR-503* and PYK2 by MPs. Moreover, the modifications observed in recipient cells were not due to the direct transfer of MP cargo but rather through regulation by transferred intermediaries. Therefore, the role of MPs in directly and indirectly mediating drug resistance and metastasis pathways leading to the development of a more aggressive phenotype warrants further investigation. Addressing such a pathway has clinical implications as it has the potential to improve the treatment of cancer.

## Author Contributions

Joyce Gong performed the experiments, analyzed the data, and wrote the manuscript. Frederick Luk analyzed the data and contributed to technical design of the experiments. Ritu Jaiswal contributed to performing the experiments. Mary Bebawy conceived and designed the experiments. All authors contributed to writing the manuscript and approved the final manuscript.

## Conflict of Interest Statement

The authors declare that the research was conducted in the absence of any commercial or financial relationships that could be construed as a potential conflict of interest.

## Abbreviations

MDR, multidrug resistance; miRNA, microRNA; MPs, microparticles; P-gp, P-glycoprotein; PYK2, proline-rich tyrosine kinase 2.
